# Barriers to postnatal care utilization during the COVID-19 pandemic: a cross-sectional study of sociodemographic and spatial factors in Mexico City

**DOI:** 10.3389/fgwh.2025.1538565

**Published:** 2025-05-30

**Authors:** Magalhi Robledo-Clemente, Juan Carlos Silva Godínez, Lucía Daniela García Montes, Jorge Valencia-Ortega, Renata Saucedo

**Affiliations:** ^1^ Hospital de Gineco-Obstetricia 3, “Dr. Víctor Manuel Espinosa de los Reyes Sánchez” Centro Médico Nacional “La Raza”, Instituto Mexicano del Seguro Social, México City, México; ^2^ Escuela Nacional Colegio de Ciencias y Humanidades, Universidad Nacional Autónoma de México, México City, México; ^3^ ECPE Department-PPCR Program, Harvard T.H. Chan School of Public Health, Boston, MA, United States; ^4^ Laboratorio de Investigación en Biología Molecular, Hospital Infantil de México “Federico Gómez”, Mexico City, Mexico; ^5^ Unidad de Investigación Médica en Enfermedades Endocrinas, Hospital de Especialidades, Centro Médico Nacional Siglo XXI, Instituto Mexicano del Seguro Social, Mexico City, Mexico

**Keywords:** postnatal care, COVID-19 pandemic, spatial analysis, healthcare access, maternal health, socioeconomic factors

## Abstract

**Background:**

The COVID-19 pandemic has disrupted maternal and postnatal care globally, particularly in low- and middle-income countries. This study investigated sociodemographic, geographic, psychosocial, and obstetric factors associated with inadequate postnatal care utilization in Mexico City during the pandemic.

**Methods:**

We conducted a cross-sectional survey among 719 postpartum women at a major obstetric hospital in Mexico City. Maternal sociodemographic data, social support (MOS survey), prenatal care quality (Kessner Index), postnatal depression (Edinburgh Scale), care satisfaction (SERVQUAL), and obstetric history were assessed. Spatial regression models evaluated associations between maternal factors, socioeconomic status (AMAI Index), and postnatal visits, incorporating geographic dependencies.

**Results:**

Significant spatial autocorrelation in postnatal visit frequency was observed (*χ*^2^ = 14.07; *p* < 0.001) indicating geographic dependencies in healthcare utilization. Higher consultation rates were associated with being a non-qualified worker (*β* = 0.252), living with a domestic partner (*β* = 0.196), and belonging to the medium-low socioeconomic group (*β* = 0.297). Maternal education showed no significant association. The spatial error term confirmed significant geographic dependencies (*β* = −0.153, *p* < 0.001).

**Conclusions:**

Geographic location, occupation, and socioeconomic status significantly influence postnatal visit frequency during public health crises, while education plays a lesser role. These findings suggest the need for targeted interventions addressing geographic barriers and incorporating mental health support to enhance maternal healthcare access among vulnerable populations. Future research should focus on developing integrated care frameworks that can better withstand disruptions during public health emergencies.

## 1 Introduction

The COVID-19 pandemic has significantly disrupted healthcare systems worldwide, with profound effects on maternal and postnatal care. These disruptions exposed and exacerbated existing vulnerabilities among postpartum women, leading to both direct and indirect consequences for maternal and neonatal health (1). The challenges encountered during this period include delayed or missed postnatal appointments, restricted access to healthcare facilities, and the implementation of social distancing measures that hindered essential care services (2). These barriers have contributed to adverse maternal and neonatal outcomes, underscoring the urgent need to address the quality of postnatal care during public health crises.

Psychosocial stressors played a critical role in shaping the postnatal experiences of women during the pandemic. Increased anxiety, depression, and a lack of social support were commonly reported among postpartum women, with studies indicating that nearly 43% of postpartum women in the United Kingdom experienced clinically significant depression and 61% faced heightened anxiety due to pandemic-related isolation (3). Similarly, research from the United States demonstrated that prenatal stress, especially during the early months of the pandemic, was associated with an increased risk of adverse perinatal outcomes, including preterm birth and low birth weight (4). These findings highlight the critical need for integrating mental health support into postnatal care frameworks, particularly during times of global crisis.

Disparities in postnatal care have been further pronounced by socioeconomic and geographic factors, which have limited access to essential services for many women. The impact of the COVID-19 pandemic on postnatal care was particularly significant in low- and middle-income countries, where healthcare infrastructure is often underdeveloped, and disparities in care are more pronounced (5). Women from lower socioeconomic backgrounds, those with limited education, and those residing in rural or remote areas were found to be significantly less likely to receive adequate postnatal care, increasing their risk of postpartum complications (6). For instance, a study in India revealed that rural women from lower socioeconomic groups were less likely to access both antenatal and postnatal care compared to their urban counterparts, reflecting broader inequities in healthcare access (7). Similarly, research conducted in Myanmar highlighted that postnatal care utilization was notably lower among women living in rural regions with limited healthcare resources, a situation further exacerbated during the pandemic (8).

In Mexico, the factors contributing to inadequate postnatal care remain under-documented, despite the significant impact on maternal health. A study conducted prior to the COVID-19 pandemic, examining six primary care clinics within the Mexican Social Security Institute (IMSS), found that only 49.5% of women attended postnatal checkups. Factors significantly associated with the lack of postnatal care included residing 5 km or more from a healthcare facility, inadequate prenatal care, and incomplete postpartum hospital care. However, the situation after the onset of COVID-19 remains undocumented, highlighting the critical need for targeted strategies to improve postnatal care access and quality, particularly in vulnerable populations (9).

Therefore, this study aims to identify the sociodemographic, geographic, psychosocial, and obstetric factors associated with inadequate postnatal care among women who delivered at the Unidad Médica de Alta Especialidad Hospital de Gineco-Obstetricia No. 3 “Dr. Víctor Manuel Espinosa de los Reyes Sánchez” in Mexico City during the COVID-19 pandemic. These critical factors are emphasized to inform targeted interventions that can reduce the negative impact of future health crises on postnatal care, particularly for vulnerable populations.

## 2 Materials and methods

### 2.1 Study design and participants

We conducted a cross-sectional survey study at the Unidad Médica de Alta Especialidad Hospital de Gineco Obstetricia No. 3 “Dr. Víctor Manuel Espinosa de los Reyes Sánchez, Centro Médico Nacional La Raza,” (CMN La Raza) of the Instituto Mexicano del Seguro Social (IMSS), located in Mexico City. This hospital was selected due to its status as one of the largest tertiary obstetric care centers within the IMSS network. It serves a highly diverse patient population throughout Greater Mexico City, encompassing urban, peri-urban, and underserved communities. The facility's substantial delivery volume and extensive catchment area rendered it an optimal site for investigating spatial disparities in postnatal care access and visit frequency. Although based on a single institution, the hospital's extensive catchment area covering diverse urban and peri-urban populations enhances the generalizability of our findings to other large metropolitan areas in Mexico.

The study targeted puerperal women who delivered at this facility during the COVID-19 pandemic. A consecutive sampling approach was employed, enrolling all eligible women who delivered at the hospital during the study period and met inclusion criteria. Eligible participants were women aged 18–49 years, in the immediate postpartum period following vaginal delivery or cesarean section, with a single live newborn. Participants were also required to be residents of Greater Mexico City (Including the States of Mexico and Hidalgo) and beneficiaries of the IMSS.

Mothers whose newborns were admitted to the pathological nursery, intermediate neonatal care units, or neonatal intensive care units, as well as to mothers requiring admission to an intensive care unit during the postpartum period were excluded from the study. Women with physical or mental conditions that prevented them from responding to the study questionnaire and those with twin pregnancies were also excluded.

Participants were further eliminated if they or their newborns were hospitalized during the follow-up period, if the mother sought postnatal care in a private setting, or in the event of the death of the mother or newborn before any of the scheduled postnatal follow-up consultations.

### 2.2 Data collection

A structured questionnaire, comprising open-ended, closed-ended, and Likert-type questions, was used to collect data on a range of variables including maternal sociodemographic characteristics, social support, postnatal depression screening, perceived quality of prenatal care, postnatal hospital care, postnatal care at their assigned primary care center (family medicine unit (FMU), and gynecological-obstetric history. Identification data were collected, with each participant assigned a unique identifier, and contact information. Trained obstetrics and gynecology medical residents administered paper-based questionnaires in a private hospital setting to maintain confidentiality and data integrity. Responses were subsequently entered into a structured database using Google Sheets (Google LLC, Mountain View, CA, USA). Following data collection, all information was exported to Stata 18 BE for comprehensive cleaning and statistical analysis.

Geographic coordinates for the FMUs were primarily obtained from the official IMSS healthcare facility directory. When coordinates were unavailable or incomplete, we used Google Maps (Google LLC, Mountain View, CA, USA) to geocode facility locations. Patient residential locations were geocoded using ZIP codes, and their centroid coordinates were retrieved through Google Maps to ensure spatial accuracy while preserving participant confidentiality. This methodology allowed for comprehensive spatial mapping and analysis of healthcare access patterns, and has been successfully applied in previous spatial healthcare research to ensure privacy while maintaining geographic precision (10, 11).

### 2.3 Measurement instruments

Several validated instruments were used to measure the study variables. The socioeconomic level was assessed using the AMAI 2018 Socioeconomic Level Index, designed by the Mexican Association of Market Research Agencies, which classifies households based on six dimensions of well-being, including human capital, infrastructure, and connectivity (12). Prenatal care quality was evaluated using the Kessner Index (13), which assesses the timing and number of prenatal visits, classifying the care as adequate, intermediate, or inadequate. Social support was measured using the Medical Outcomes Study Social Support Survey (MOS) (14), which evaluates emotional, instrumental, and positive social interactions. For the purposes of the analyses, the subscales of the MOS were standardized to a score of 100%, allowing for consistent comparisons across the different dimensions of social support.

Postnatal depression was screened using the Edinburgh Postnatal Depression Scale (15), a widely used tool that helps identify depressive symptoms in postpartum women. Satisfaction with prenatal care was assessed through a modified SERVQUAL survey, including 22 questions across five dimensions: Reliability, Responsiveness, Safety, Empathy, and Tangibles. Scores are categorized from “Extremely Poor” to “Extremely Good” based on the total score from the individual items (16).

### 2.4 Predictor and outcome variables

The primary predictor variables included socioeconomic level, perceived social support, prenatal care quality, and postnatal depression. Sociodemographic characteristics such as age, education, marital status, and employment were also considered. The primary outcome was inadequate postnatal care, determined by number of visits to scheduled postnatal visits and perceived quality of care during the postpartum period. Multivariable analyses adjusted for covariates including maternal health status, neonatal health, and healthcare access during the pandemic to evaluate their association with inadequate postnatal care.

### 2.5 Statistical analysis

For data analysis, a descriptive examination of the clinical attributes, and sociodemographic characteristics was conducted. Means and standard deviations (SD) were used for continuous variables, while counts and percentages were used for categorical variables. Missing data were assessed, and complete case analysis was conducted as missingness was under 5% for all included variables. T-tests and Chi-square tests were used to evaluate differences in baseline characteristics between the two groups.

**Figure 1:**
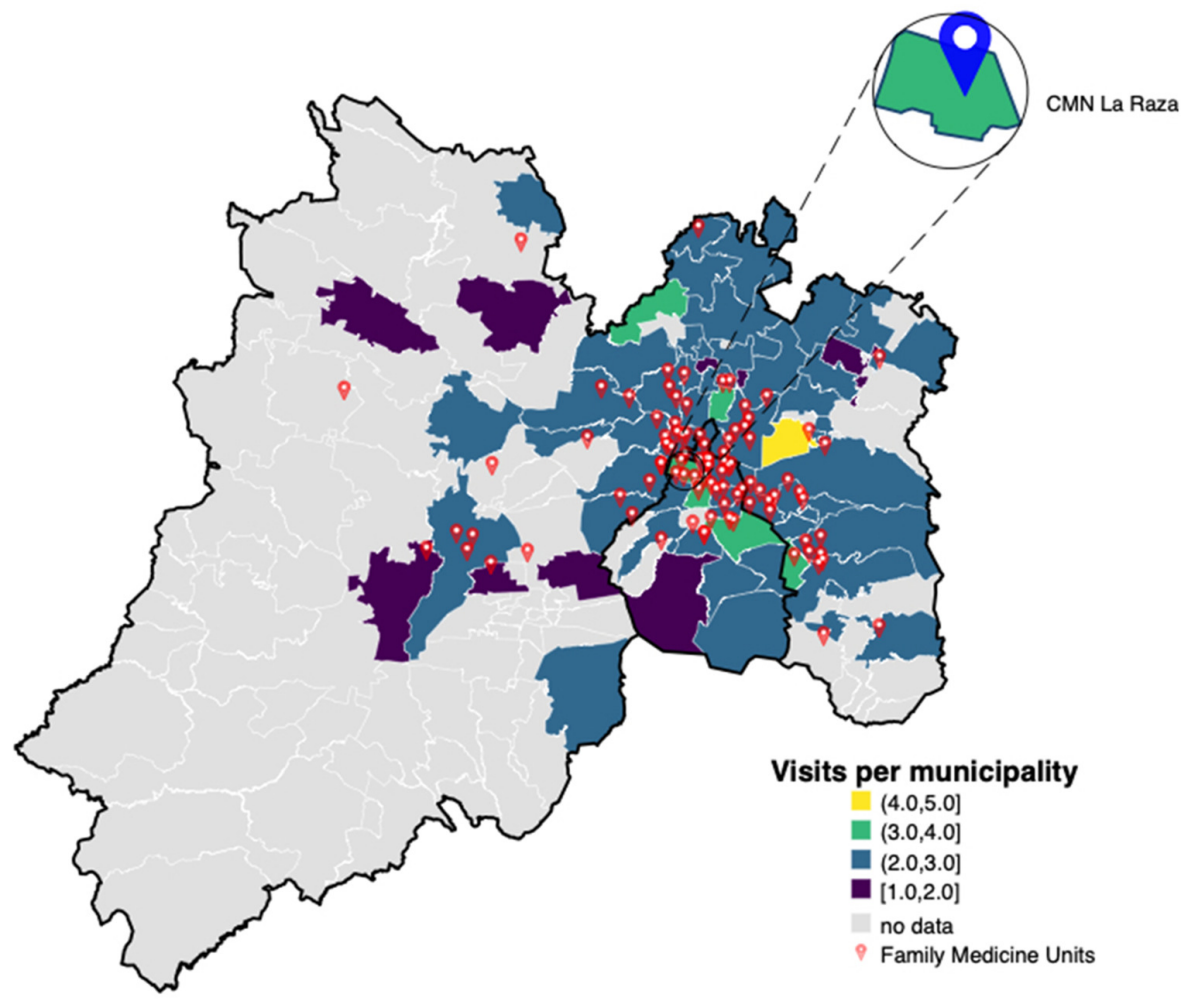
Choropleth map of average post-delivery consultations by municipality. The map displays the average number of post-delivery consultations for newborns across municipalities within Greater Mexico City, including both Mexico City and parts of Mexico state. A color scale is included, with darker shades indicating lower average consultation frequencies and lighter shades indicating higher frequencies. Red dots indicate the locations of Family Medicine Units, with an inset highlighting CMN La Raza as a reference point. The spatial distribution suggests clustering of consultation rates, implying that geographic proximity to healthcare facilities may influence the frequency of post-delivery care.

A choropleth map was utilized to visually assess spatial relationships between the number of visits and patient localization, providing a detailed representation of the geographic distribution of postnatal visit frequency (see Figure 1). Geospatial data aggregation was performed at the municipality and FMU levels, focusing on municipalities within Mexico City and the State of Mexico. To evaluate spatial autocorrelation in the regression residuals, Moran's I test was conducted, helping identify spatial patterns in postnatal visit frequency across these regions.

A Spatial Durbin Model (SDM) was employed to analyze associations between maternal characteristics, socioeconomic factors, and the average frequency of postnatal visits to FMUs. Model fit was assessed using pseudo Akaike Information Criterion (AIC) and pseudo Bayesian Information Criterion (BIC) values, both derived from the residual sum of squares (RSS), calculated as AIC = *n* × ln (RSS/*n*) + 2*k* and BIC = *n* × ln (RSS/*n*) + *k* × ln (*n*), where *n* denotes the number of observations and *k* the number of estimated parameters. This model was selected based on its superior analytical performance and comprehensive capacity to adjust for spatial dependencies, as indicated by lower pseudo AIC and BIC values compared to alternative specifications. By integrating features of both the Spatial Lag Model (SLM) and the Spatial Error Model (SEM), the SDM allows for simultaneous adjustment for spatial autocorrelation in both dependent and independent variables, thereby capturing unobserved spatial influences that could bias simpler Ordinary Least Squares (OLS) or lag-only models. The spatial structure was defined using an inverse-distance weighting matrix based on geographic coordinates of patients and their corresponding FMUs, effectively accounting for spatial clustering. Spatial lag terms captured the influence of neighboring municipalities, while spatial error terms adjusted for location-specific unmeasured effects. This approach offered a robust comparative framework for model evaluation, despite the recognized limitations of traditional information criteria when applied to Generalized Spatial Two-Stage Least Squares (GS2SLS) estimators. Statistical significance was defined as a *p*-value less than 0.05. All analyses were performed using Stata 18 BE (StataCorp, College Station, TX, USA).

## 3 Results

A total of 719 recently delivered women were included in the analysis. The mean age of participants was 30.78 years (SD = 12.57). Mean number of consultations received by all participants was 2.76 (95% CI: 1.72–3.80). Most participants were housewives (37.41%). Among the employed participants, semi-qualified workers were the most frequent group, comprising 27.54% of the employed population. The highest level of education was high school/technical, accounting for 45.48% of the study population. The median number of previous pregnancies was 2 (range: 1–9), and the median gestational age at delivery was 38.7 weeks. Notably, 201 women (27.96%) screened positive for depression, indicating a significant prevalence of this condition. Social Support, Emotional Well-being, and Quality of Care were evaluated on a standardized 100% scale, with Social Support scoring an average of 91.52 (SD = 12.66), Emotional Well-being 40.45 (SD = 17.62), and Quality of Care 86.51 (SD = 21.55), reflecting high perceived social support and variability in emotional health.

Socioeconomic status was categorized into six distinct groups. The “High” category was the most prevalent, representing 36.02% of participants, followed by “Medium-high” at 31.29%. The “Medium” and “Medium-low” groups comprised 20.03% and 10.29%, respectively. A small minority fell into the “Low” (1.67%) and “Low-high” (0.70%) categories.

Prenatal care adequacy, assessed using the Kessner Index, showed that 71.35% of women received adequate care, 26.98% received intermediate care, and 1.67% received inadequate care, underscoring areas needing improvement. Table 1 presents the demographic characteristics stratified by the Kessner Index.

**Table 1:** Demographic characteristics or participants. Data are presented as mean (SD) for continuous measures, and *n* (%) for categorical measures.

Variable	Kessner adequacy index			*p*-value
	Inadequate	Intermediate	Adequate	
	*N* = 12	*N* = 194	*N* = 513	
Maternal age (years)	36 (6)	31 (10)	31 (14)	0.31
Maternal occupation				0.092
Housewife	5 (41.7%)	67 (34.5%)	197 (38.4%)	
Non-qualified worker	3 (25.0%)	25 (12.9%)	62 (12.1%)	
Semi-qualified worker	1 (8.3%)	46 (23.7%)	151 (29.4%)	
Qualified worker	2 (16.7%)	42 (21.6%)	90 (17.5%)	
Student	1 (8.3%)	10 (5.2%)	10 (1.9%)	
Other	0 (0.0%)	4 (2.1%)	3 (0.6%)	
Educational level				0.64
None	0 (0.0%)	1 (0.5%)	7 (1.4%)	
Elementary school	0 (0.0%)	2 (1.0%)	5 (1.0%)	
Junior-high school	1 (8.3%)	25 (12.9%)	60 (11.7%)	
High school/Technical	6 (50.0%)	87 (44.8%)	234 (45.6%)	
College	5 (41.7%)	67 (34.5%)	194 (37.8%)	
Graduate	0 (0.0%)	12 (6.2%)	13 (2.5%)	
Socioeconomic level				0.99
High	5 (42%)	73 (38%)	181 (35%)	
Low	0 (0%)	2 (1%)	10 (2%)	
Low-high	0 (0%)	2 (1%)	3 (1%)	
Medium	2 (17%)	37 (19%)	105 (20%)	
Medium-high	3 (25%)	60 (31%)	162 (32%)	
Medium-low	2 (17%)	20 (10%)	52 (10%)	
Gestational age	39 (0)	39 (1)	39 (1)	0.29
Number of previous pregnancies	2 (1)	2 (1)	2 (1)	0.64
Number of previous deliveries	1 (1)	1 (1)	1 (1)	0.040
Number of previous c-sections	1 (1)	1 (1)	1 (1)	0.11
Number of previous abortions	0 (0)	0 (1)	0 (1)	0.060
Presence of comorbidities during pregnancy				<0.001
No	3 (25.0%)	64 (33.0%)	87 (17.0%)	
Yes	9 (75.0%)	130 (67.0%)	426 (83.0%)	
Gestation-induced hypertension				0.75
No	11 (91.7%)	173 (89.2%)	446 (86.9%)	
Yes	1 (8.3%)	21 (10.8%)	67 (13.1%)	
Gestational diabetes				0.007
No	7 (58.3%)	139 (71.6%)	303 (59.1%)	
Yes	5 (41.7%)	55 (28.4%)	210 (40.9%)	
Thyroid disease				0.96
No	9 (75.0%)	140 (72.2%)	366 (71.3%)	
Yes	3 (25.0%)	54 (27.8%)	147 (28.7%)	
Anemia				0.42
No	10 (83.3%)	179 (92.3%)	468 (91.2%)	
Yes	2 (16.7%)	15 (7.7%)	45 (8.8%)	
Socioeconomic level				0.99
Low	0 (0.0%)	2 (1.0%)	10 (1.9%)	
Low-high	0 (0.0%)	2 (1.0%)	3 (0.6%)	
Medium	2 (16.7%)	37 (19.1%)	105 (20.5%)	
Medium-high	3 (25.0%)	60 (30.9%)	162 (31.6%)	
Medium-low	2 (16.7%)	20 (10.3%)	52 (10.1%)	
High	5 (41.7%)	73 (37.6%)	181 (35.3%)	
Medical outcomes				
Social support	95 (4)	92 (10)	91 (14)	0.32
Emotional well-being	34 (15)	40 (17)	41 (18)	0.40
Quality of care	93 (12)	87 (20)	86 (22)	0.57

### 3.1 Spatial analysis and regression findings

Spatial clustering analysis using Moran's I statistic revealed significant spatial autocorrelation in consultation frequency by maternal location io (*χ*^2^ = 14.07, *p* < 0.001). This finding suggested that geographical factors significantly influence the frequency of post-delivery consultations.

The spatial regression analysis indicated that maternal occupation significantly influenced postnatal visit frequency, with non-qualified workers having more consultations compared to housewives (*β* = 0.252, 95% CI: 0.007–0.497, *p* = 0.044). Marital status was also significant, with domestic partners associated with increased consultations (*β* = 0.196, 95% CI: 0.024–0.368, *p* = 0.025). No significant associations were found between maternal education levels and postnatal visit frequency, with coefficients ranging from −0.655 for elementary school education (95% CI: −1.768 to 0.458, *p* = 0.249) to −0.063 for graduate-level education (95% CI: −0.927 to 0.800, *p* = 0.886). The results of the spatial regression analysis are presented in Table 2.

**Table 2:** Spatial regression analysis.

Variable	Coefficient	SE	*p*-value	95% CI
Distance to FMU	0.0009772	0.0076834	0.899	−0.01408 to 0.01604
Maternal age	0.0038477	0.0030053	0.2	−0.00204 to 0.00974
Non-qualified worker	0.2519589	0.1251353	0.044^a^	0.00670 to 0.49722
Other occupation	−0.2560734	0.3885044	0.51	−1.01753 to 0.50538
Marital status				
Domestic partnership	0.1962265	0.087709	0.025^a^	0.02432 to 0.36813
Divorced	−0.0191296	0.2886523	0.947	−0.58488 to 0.54662
Single	0.0981671	0.1687335	0.561	−0.23254 to 0.42888
Educational level				
Elementary school	−0.6551566	0.5679941	0.249	−1.76841 to 0.45809
Junior-high school	−0.2424305	0.3931383	0.537	−1.01297 to 0.52811
High school/technical	−0.1453815	0.376871	0.7	−0.88404 to 0.59327
Number of pregnancies	−0.0093251	0.0479587	0.846	−0.10332 to 0.08467
Number of childbirths	−0.0172555	0.0690203	0.803	−0.15253 to 0.11802
Number of C-sections	−0.0116446	0.07266	0.873	−0.15406 to 0.13077
Presence of comorbidities	0.1543548	0.0943969	0.102	−0.03066 to 0.33937
Medical outcomes				
Social support	0.0025054	0.0030466	0.411	−0.00347 to 0.00848
Emotional well-being	−0.0059359	0.0036204	0.101	−0.01303 to −0.00116
Quality of care	−0.000964	0.001837	0.6	−0.00456 to 0.00264
Depressive symptoms	0.2499202	0.1425901	0.08	−0.02955 to 0.52939
Socioeconomic level				
Low	−0.3606414	0.3393078	0.288	−1.02567 to 0.30439
Medium-low	0.2966964	0.144044	0.039	0.01438 to 0.57902
Spatial error term	−0.1526152	0.0412268	<0.001^a^	−0.23342 to −0.07181

^a^ The variables “social support,” “emotional well-being,” and “quality of care” are derived from the Medical Outcomes Study (MOS) and have been standardized to a 100% score. SE, standard error, Distance to FMU is measured in kilometers.

Other covariates, including maternal age, number of pregnancies, number of childbirths, and number of cesarean sections, were not significantly associated with the outcome variable. Although comorbidities (*β* = 0.154, 95% CI: −0.031 to 0.339, *p* = 0.102) and emotional well-being scores (*β* = −0.006, 95% CI: −0.013 to 0.001, *p* = 0.101) did not reach statistical significance, their coefficients suggest potential effects that merit further investigation. Socioeconomic level analysis revealed that individuals from the “Medium-low” socioeconomic group had a statistically significant positive association with postnatal visit frequency (*β* = 0.297, 95% CI: 0.014–0.579, *p* = 0.039). The significant spatial error term (*p* < 0.001) highlighted the presence of spatial dependencies within the model's residuals.

Model goodness-of-fit was assessed using pseudo AIC and pseudo BIC calculations. The full spatial lag + error model achieved a pseudo AIC of 47.31 and a pseudo BIC of 184.39, compared to the spatial lag-only model (pseudo AIC = 47.38, pseudo BIC = 189.04). These results suggest that the full spatial lag and error model offered the best balance between model complexity and explanatory power.

## 4 Discussion

This study identified critical sociodemographic, geographic, psychosocial, and obstetric factors associated with inadequate postnatal care among women who delivered at a major obstetric hospital in Mexico City during the COVID-19 pandemic. Our findings indicate that inadequate postnatal visit frequency is significantly influenced by spatial factors, maternal occupation, marital status, and socioeconomic level. These results highlight the need for targeted interventions to enhance access to postnatal care, especially among vulnerable populations.

Our study offers valuable insights into the determinants of postnatal visit frequency during a global health crisis, expanding upon previous research that has identified similar barriers. Prior evidence has shown that the COVID-19 pandemic disrupted maternal healthcare services worldwide, resulting in missed or delayed postnatal visits and heightened maternal stress and anxiety due to restricted access to healthcare facilities (1). Our findings build on this evidence by focusing specifically on the Mexican context, where inadequate postnatal care was linked to geographic location of healthcare facilities, lower socioeconomic status, and psychosocial factors such as depression and perceived social support. These results align with studies from other low- and middle-income countries, where rural residence, lower education, and socioeconomic barriers have been identified as key determinants of reduced postnatal care uptake (6).

It is important to recognize that women's family and household circumstances vary, and decisions about who is involved in postnatal care should be left to the mother. The role of the baby's father or other parental figures is also crucial in the baby's care. Recognizing these dynamics is essential when planning postnatal interventions, as family involvement can influence care adherence and outcomes.

In addition, it is important to recognize that women with mental health concerns, or those worried they might have them, may be reluctant to disclose or discuss their issues due to fears of stigma, negative perceptions of their ability as mothers, or anxiety about their baby being taken into care. Such women may also be hesitant to engage in, or may struggle to participate in, treatment due to avoidance behaviors related to their mental health issues or substance use (17). These psychosocial barriers must be considered when designing interventions aimed at increasing postnatal visit frequency.

Moreover, the American College of Obstetricians and Gynecologists advises that postnatal care should be individualized, recommending a comprehensive evaluation for all women within 12 weeks postpartum (18). The provision of individualized care often involves multiple professionals, underscoring the importance of a multidisciplinary approach to postnatal healthcare, especially in vulnerable populations. In line with this recommendation, our findings suggest that tailored interventions may be critical for improving postnatal care uptake, particularly among women facing geographic and socioeconomic challenges.

In Mexico, the factors contributing to inadequate postnatal care remain under-documented, despite the significant impact on maternal health. A study examining six primary care clinics within the IMSS found that only 49.5% of women attended postnatal checkups. Factors significantly associated with the lack of postnatal care included residing 5 km or more from a healthcare facility, inadequate prenatal care, and incomplete postpartum hospital care, highlighting the critical need for targeted strategies to improve postnatal care access and quality, particularly in vulnerable populations. These findings further align with regional literature from Mexico and Latin America, where geographic location, transportation barriers, and fragmentation in healthcare systems have been shown to significantly limit maternal and postnatal care access. For instance, a study conducted in Mexico City found that women residing more than 5 km from their assigned clinic were significantly less likely to attend postnatal follow-up appointments, even after adjusting for education and income levels (9). Similarly, research from Guatemala and Peru has highlighted the role of inadequate infrastructure, lack of culturally sensitive services, and logistical challenges in reducing utilization of maternal health services in rural and peri-urban settings (19, 20). Incorporating these structural and contextual factors is essential for understanding regional disparities in maternal care.

In this study, spatial analysis revealed significant geographic clustering of postnatal visits, emphasizing the importance of localization rather than mere distance to healthcare facilities. While distance has been widely reported as a barrier to maternal care (9), our findings suggest that spatial clustering and other location-specific dynamics may be more relevant than distance alone. This is supported by the spatial regression model, where the significant error lag term reflects unmeasured contextual influences at the local level. This highlights the complexity of spatial dependencies and the need to consider broader contextual elements when addressing barriers to maternal healthcare access. The selection of the spatial lag + error model was supported by model fit criteria, highlighting the importance of accounting for unobserved spatial dependence when analyzing average postnatal visit frequency. In addition, our spatial regression model showed significant associations between maternal occupation and consultation frequency, with non-qualified workers more likely to attend postnatal visits compared to housewives. These findings support the notion that employment-related factors, including access to health insurance and logistical resources, facilitate postnatal care attendance, particularly given that IMSS coverage is closely tied to formal employment (21). Formally employed individuals are automatically enrolled in IMSS through payroll contributions, granting them and their dependents access to primary and specialized care. This structural linkage means that employment status directly influences healthcare access. Conversely, those engaged in informal employment or unemployed often lack such coverage, leading to disparities in postnatal visit frequency. Our findings suggest that women with formal employment may have more consistent access to postnatal care services due to their integration into the IMSS system, highlighting the importance of employment status in healthcare accessibility (22). In contrast, educational attainment did not show a significant association with postnatal visit frequency in our study.

One possible explanation is that, in this population, even educated women may encounter constraints such as limited appointment availability, long distances, or competing household responsibilities (21). Moreover, educational attainment does not always translate into higher health literacy or ability to navigate IMSS services, as shown in both international and Mexican studies (23, 24). These patterns echo findings from Latin America and Asia, where structural and financial barriers often outweigh the protective effects of formal education (7, 8). Additionally, potential misclassification or residual confounding related to socioeconomic variables may have attenuated the observed association between education and postnatal visit frequency.

However, the lack of significant associations between maternal education and postnatal visit frequency in our study contrasts with some previous reports, suggesting that in our setting, geographic and financial barriers may outweigh the benefits of higher education in facilitating healthcare access (25). This discrepancy may reflect structural limitations within the Mexican healthcare system that affect all women, regardless of their educational background. For example, a qualitative study conducted in Chiapas, Mexico, found that even highly educated women encountered obstacles such as transportation costs, cultural norms, and a low perceived need for postnatal care, all of which reduced service utilization (26). This supports the notion that formal education does not always translate into health literacy or effective system navigation. Additionally, a national-level analysis of maternal and newborn care in Mexico underscored deficiencies in care quality and content, further supporting the idea that education alone does not ensure better outcomes (27). Together, these insights highlight the need to address systemic and contextual barriers that persist independently of educational attainment.

The significant prevalence of depressive symptoms among women attending postnatal care attendance found in our study aligns with observations from high-income settings during the pandemic, where elevated anxiety and depression levels among postpartum women were common (3). The high prevalence of depression among our study participants further emphasizes the importance of integrating mental health support into postnatal care frameworks, particularly during crises when psychosocial stressors are heightened.

Our study has some limitations that should be acknowledged. First, as an observational study based on survey data, causality cannot be firmly established, and the findings are susceptible to biases inherent in self-reported data. Additionally, selection bias may be present due to the hospital-based recruitment strategy, and recall bias could have influenced responses regarding past care experiences. The inclusion of participants from diverse localities introduces variability in healthcare access, socioeconomic status, and cultural practices that were not fully adjusted for in the analysis. Geographic proximity to healthcare facilities, although measured, does not fully capture other barriers such as transportation availability, clinic capacity, or personal preferences, which may also affect post-delivery visit frequency. Moreover, the observed spatial clustering may reflect unmeasured confounders, limiting the generalizability of our findings beyond the study population.

Further research is needed to explore how interventions that address geographic barriers, such as mobile health units or community-based outreach, can enhance postnatal care uptake in under-resourced settings. Longitudinal studies would be valuable in assessing the long-term impact of pandemic-related disruptions on maternal and neonatal health outcomes, particularly among socioeconomically disadvantaged groups.

## 5 Conclusion

This study emphasizes the need to address both spatial and psychosocial barriers to improve postnatal care access in Mexico, particularly during public health emergencies. It is also essential to recognize that vulnerable women often face multiple complex social factors simultaneously—such as poverty, unemployment, and incarceration—that can influence their care needs. Future research should evaluate targeted interventions, such as telemedicine and enhanced mental health support, to mitigate the impact of geographic and socioeconomic disparities on postnatal care. Policymakers should consider expanding healthcare access points in underserved areas and developing strategies to support postpartum women experiencing psychological distress. For example, IMSS could consider expanding mobile FMUs in underserved municipalities and supporting postpartum women in informal employment through targeted outreach programs and simplified enrollment procedures.

Key factors associated with inadequate postnatal care are identified, providing a foundation for evidence-based interventions to address the unique challenges faced by vulnerable populations during global health crises. In addition, healthcare professionals should consider the individual needs of each woman, acknowledging that their requirements may extend beyond population-level solutions and involve input from multiple sectors, including health and social care professionals. Strengthening maternal healthcare systems to withstand future emergencies will be critical to protecting the health and well-being of postpartum women and their infants.

## Data Availability

The raw data supporting the conclusions of this article will be made available by the authors, without undue reservation.
